# The compositional landscape of minicircle sequences isolated from active lesions and scars of American cutaneous leishmaniasis

**DOI:** 10.1186/1756-3305-6-228

**Published:** 2013-08-07

**Authors:** Eduardo Henrique Gomes Rodrigues, Fábia Carla da Silva Soares, Roberto Pereira Werkhäuser, Maria Edileuza F de Brito, Octavio Fernandes, Frederico G Coutinho Abath, Adeilton Brandão

**Affiliations:** 1 Laboratório de Imunopatologia e Biologia Molecular, Departamento de Imunologia, Centro de Pesquisas Aggeu Magalhães, Fundação Oswaldo Cruz, Recife, PE, Brazil; 2 Laboratório Interdisciplinar de Pesquisas Médicas, Instituto Oswaldo Cruz, Fundação Oswaldo Cruz, Rio de Janeiro, RJ, Brazil

**Keywords:** American cutaneous leishmaniasis, Kinetoplast DNA, Minicircle, Nucleotide, Compositional bias

## Abstract

**Background:**

American cutaneous leishmaniasis (ACL) is characterized by cutaneous lesions that heal spontaneously or after specific treatment. This paper reports on the analysis of kDNA minicircle sequences from clinical samples (typical lesions and scars) that were PCR-amplified with specific primers for *Leishmania* species of the subgenus *Viannia*.

**Methods:**

From 56 clinical isolates we obtained a single amplified fragment (ca. 790 bp), which after cloning and sequencing resulted in 290 minicircle sequences from both active lesions and scars. We aimed to get a compositional profile of these sequences in clinical samples and evaluate the corresponding compositional changes. Sequences were analyzed with the compseq and wordcount (Emboss package) to get the composition of di-, tri-, tetra-, penta- and hexanucleotides. Additionally, we built a nucleotide dictionary with words of 7, 8, 9 and 10 nucleotides.

**Results:**

This compositional analysis showed that minicircles amplified from active cutaneous lesions and scars have a distinct compositional profile as viewed by nucleotide composition of words up to 10mer. With regard to the most frequent nucleotide words above length 6, there is also a distinct pattern for 7, 8, 9 and 10mer.

**Conclusion:**

These results indicate that minicircle sequences can be monitored upon direct exposure to a selection/stressing environment (e.g. chemical action) by evaluating their nucleotide compositional profile. It might be useful as a molecular tool in research concerning the evolution of infecting *Leishmania* in both vector and vertebrate hosts.

## Background

American cutaneous leishmaniasis (ACL) is a zoonotic disease caused by *Leishmania* (*Viannia*) spp., *L.* (*Leishmania*) *amazonensis*/*mexicana* or *L*. (*L*.) *infantum*, a protozoan parasite which infects the vertebrate host after being bitten by infected phlebotomus insects of the genus *Lutzomya*. Usually, human infections are either unapparent or display a clinical spectrum ranging from localized, sometimes self-healing cutaneous lesions to severe, mutilating mucocutaneous lesions to diffuse cutaneous leishmaniasis in the patients [1]. Infections caused by *Leishmania* (*V*.) spp. present typical aspects in human tissue that can be distinguished from other forms of leishmaniasis by its chronicity, latency and metastasis, resulting in migrating lesions with potential for mucosal involvement [2]. There is evidence of pathogen persistence after clinical cure of the disease. *Leishmania* (*V*.) spp. DNA can be detected in human scars [3-6] suggesting that persistence of parasites is the rule, rather than the exception.

In experimental cutaneous leishmaniasis, it is possible to show the presence of live parasites in certain strains of mice upon clinical cure by chemotherapy [7,8]. Some immunological and metabolic aspects have been related to the persistence of this parasite [3-6,9-13]. However, detailed information about parasite persistence and the genotypic nature of *Leishmania* have not been reported so far. In the nineties, the work of Karlin and Mrázek [14] demonstrated that the nucleotide composition of a particular species is biased toward some of the sixteen possible dinucleotides. This bias can be viewed as a peculiar genome signature and under certain assumptions the dinucleotide bias might reveal evolutionary distance [14]. Though the methods for nucleotide compositional analysis were developed mainly for nuclear genomes they might be used in any DNA segment as long as there is enough variability in the sequences. That is the case for trypanosomatid mitochondrial DNA, also known as kinetoplast DNA (kDNA). kDNA is composed of two kinds of molecules: large and low-copy number molecules called maxicircles and small, high-copy ones denominated minicircles. The last ones are highly variable in nucleotide composition [11] and are not easily alignable. Some studies have reported on differences in both the number of classes and frequency of each class in minicircle molecules from several kinetoplastid species [15-23].

In nuclear genomes, heterogeneities are observed for the distribution of AT and GC content [24]. Considering the clinical evolution of *Leishmania* (*V*.) spp. infection and its therapeutic practice, a question we might ask is, “What is the association between groups of words from minicircle molecules and the parasite population upon exposure to the drug?” To address this question we used the compositional profile of amplified minicircle sequences as an appropriate tool. We show here the results of such a compositional analysis of *Leishmania* (*V*.) spp. minicircle sequences from ACL patients (typical lesions and scars) coming from endemic regions in Pernambuco state, Brazil. We analyzed the composition of dinucleotides to hexanucleotides, as well as the nucleotide words from 7- up to 10mer considered to be the most frequent ones for that particular isolate, leading to a compositional bias dictionary.

## Methods

### Study area and patients

A total of 56 cutaneous biopsy specimens were obtained from two groups of subjects: 29 patients with confirmed ACL and 27 patients clinically cured. Both groups come from the Amaraji Municipality and neighboring regions in Pernambuco state, Brazil, a region where *Leishmania* (*V*.) spp. is endemic. The first group was composed of biopsy specimens from patients before treatment, while the second group was obtained from patients clinically cured of ACL after receiving chemotherapy by meglumine antimonite (10 mg/kg/day intramuscularly for 20 days, repeated if necessary). The project was approved by the ethics committee of Centro de Pesquisas Aggeu Magalhães (CPqAM/FIOCRUZ) (No. 16/01), and all enrolled subjects provided written consent. The definition of a confirmed case in the group of patients clinically cured of ACL was as follows: (i) a previous diagnosis of ACL based on clinical and epidemiological evidence (i.e., the presence of typical lesions, compatible epidemiological history, and clinical response to specific treatment), microscopic smear examination, histopathological examination, isolation by axenic culture, or detection of circulating antibodies by indirect immunofluorescence (IIF); (ii) healing of lesions with the presence of scar for at least 6 months; and (iii) the absence of lesions suggestive of active disease or relapse.

### Patient samples

Samples were collected by skin-punch biopsy and consisted of 4–6 mm diam. specimens at the border of the lesion under sterile conditions and local anesthesia (3% prilocaine chloridrate). All specimens were stored at −20°C for further processing for Polymerase Chain Reaction (PCR). These samples were collected from 1995 to 2000 in the field or at the outpatient facility of a reference hospital (Hospital das Clínicas, Universidade Federal de Pernambuco-UFPE, Recife).

### Extraction of DNA and PCR amplification

DNA was purified by using the Genomic Prep Cells and Tissue DNA isolation kit (GE Life Sciences) according to the manufacturer’s instructions. Approximately 20 mg of frozen tissue samples were used for each DNA isolation. After purification, the DNA was suspended in 100 μL of TE (10 mM Tris, 1 mM EDTA [pH 8.0]) and stored at −20°C until use. A PCR-based system specific for *Leishmania* (*Viannia*) was used with the primers LEIB1 (5′-GGG GTT GGT GTA ATA TAG TGG-3′) and LEIB2 (5′-CTA ATT GTG CAC GGG GAG G-3′) [25]. A 25 μL PCR mixture was prepared containing 10 mmol/L Tris–HCl, 50 mmol/L KCl, 0.1 mg/mL gelatin, 1.5 mmol/L MgCl_2_, 0.2 mmol/L each dNTP, 25 pmol of each primer, 2.5 U of *Taq* DNA polymerase (GE Life Sciences), and 2 μL of the purified DNA. The thermal regime consisted of annealing at 65°C for 1 min, extension at 72°C for 1 min, and denaturation at 94°C for 1 min, for 35 cycles. Tubes were heated for 4 min at 94°C before cycling. Several negative controls (no DNA) and positive controls (100 or 10 pg of *L*. *braziliensis* genomic DNA [IOC-L-566-MHOM/BR/75/M2903]) were included for every PCR. Amplification was carried out on a Perkin-Elmer model 4800 thermocycler.

The amplified fragments (10 μL) were separated by electrophoresis at 6 V/cm in agarose gels in 1X TAE (40 mM Tris-Acetate, 1 mM EDTA). Ethidium bromide-stained gels were visualized and photographed under UV light. The *Leishmania* (*Viannia*)-specific PCR amplifies a 750 bp fragment and is able to detect ca. 10 fg of promastigote genomic DNA [1]. This amplicon is unique to this subgenus and represents a single linearized minicircle.

### Cloning and sequencing

The amplified minicircles, as described above, were purified using SephaglasTM BandPrep Kit (GE Life Sciences) and cloned into pCR 4 TOPO TA vector (TOPO TA Cloning Kit for Sequencing (Invitrogen Life Technologies, California, USA) according to the manufacturer’s instructions. The TOP10 strain of *Escherichia coli* (Invitrogen Life Technologies, California, USA) were transformed and 10 recombinant colonies for each sample were selected, the plasmid purified by standard procedures, and further digested with *Eco* RI to confirm the presence of an insert [26].

Selected plasmids were further purified with SephaglasTM BandPrep Kit (GE Life Sciences) according to the manufacturer’s instructions. Sequencing reactions were carried out with primers T3 (5′–ATT AAC CCT CAC TAA AGG GA–3′), T7 (5′–TAA TAC GAC TCA CTA TAG GG–3′), M13 Forward (5′–GTA AAA CGA CGG CCA G–3′) and M13 Reverse (5′–CAG GAA ACA GCT ATG AC–3′) using the BigDye Terminator v3.1 Cycle Sequencing Kit. Sequencing fragments were purified with EtOH/EDTA precipitation and electrophoresed on a DNA-ABI Prism 3730 automated sequencer (Applied Biosystems).

### Data analysis

Minicircle raw sequences were edited and then aligned with MEGA version 3.1 [27]. The composition of the di-, tri-, tetra-, penta- and hexanucleotides were obtained with compseq (http://www.emboss.bioinformatics.nl/cgi-bin/emboss/compseq). The most frequent words between seven and ten nucleotides were extracted with Wordcount (http://emboss.bioinformatics.nl/cgi-bin/emboss/wordcount). The nucleotide word clouds were obtained using the word cloud generator at http://worditout.com/. To get the clouds we took into account that nucleotide sequences exhibit composition bias either to AT or GC and a particular nucleotide word may appear at unexpectedly high frequency. Since it is not very informative to put all the nucleotide words in a single cloud we selected only the nucleotide words in the top 10% of the frequency distribution, which includes those of an observed high count. Then, we formulated in text files the lists for words of 7, 8, 9 and 10 bases from the 10th percentile for each minicircle set and submitted them to the Worditout server. Statistics and additional graphics were generated by the statistical package PAST [28] and OpenOffice calc (http://www.openoffice.org). Nucleotide sequences reported in the paper are available in the GenBankTM database under accession numbers EF618746 to EF619032.

## Results

In this study, we analyzed 56 biopsies from patients with ACL. Twenty-nine biopsies (51.8%) met the diagnostic criteria for ACL described above and were therefore considered to be true cases of ACL and 27 (48.2%) out of these patients had scars suggestive of previous cutaneous leishmaniasis. Upon amplification and cloning, we obtained 558 rough sequences from both active lesions and scars. Further analysis by multiple alignments refined this number to the actual 290 non-redundant sequences: 175 (60.3%) from active lesions and 115 (39.7%) from scar lesions. Only minicircle sequences that showed the three conserved blocks (CSB-1, CSB-2 and CSB-3) were considered for analysis. The block CSB-3 is the site for the universal minicircle sequence [29], the 12-mer sequence 5′-GGG GTT GGT GTA A-3′, which has been considered to be the minicircle origin of replication [29,30].

### Multiple alignments of minicircle sequences

A total of 290 complete minicircles were obtained from the cloned fragments and they displayed length in the range 518 bp to 797 bp. Although minicircle sequences are not easily alignable, the raw alignment might be visualized as clusters, which reflect the compositional bias towards AT-rich segments and allow groups of minicircles to be classified into classes of high-level similarity. Sequences obtained from the same sample displayed heterogeneity that gave rise to intra-clusters. However, in comparison to sequences from different samples, all minicircles from one particular sample could be integrated into a single cluster. Thus, according to our observations, cluster majority minicircle sequences obtained from scars were grouped forming a single class, indicating some degree of homogeneity with a clonal frequency of 83/110. The minicircle sequences analyzed here fall into two main clusters: one composed of sequences obtained from 27 cloned lesion fragments and 83 scars) and another one encompassing sequences from 148 clones obtained from lesions and 32 clones from scars. We denominated these clusters as II and I, respectively. Cluster II show a preferential distribution of minicircle sequence from scar samples (83/110–75%) (Table 1) (*x*^2^ = 92.5188, df = 1 Pearson, p < 1 × 10–15). A 5% threshold of significance was chosen in the comparison between two groups [31].

**Table 1:** Analysis of minicircle groups from ACL (human lesions and scars)

**Group**	**Human lesions**	**Human scars**	**Total**
I	148	32	180
II	27	83	110
Total	175	115	290

### Distribution of nucleotides in ACL

The most frequent words in minicircle sequences from clinical samples are those containing the bases A and T. This AT bias is constant throughout words up to 10mer. One interesting feature of this bias is that the most frequent ones are present in all minicircles obtained from clinical samples in ACL (Table 2).

**Table 2:** Absolute values of adenine/thymine and cytosine/guanine of minicircle groups from ACL (human lesions and scars)

	**A + T**	**%**	**C + G**	**%**	**Total**
Human lesions	91.528	72	35.532	28	127.060 (100%)
Human scars	60.039	72.4	22.950	27.6	82.989 (100%)
Total	151.567		58.482		210.049 (100%)

### Compositional bias of the di-, tri-, tetra-, penta-, and hexanucleotide in *L. (V.) braziliensis*

**Figure 1:**
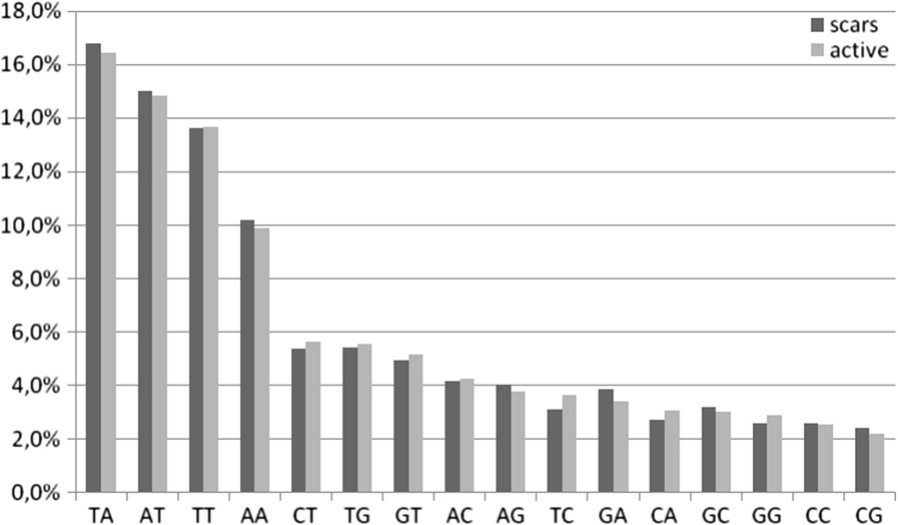
**Dinucleotide frequencies of minicircles from *Leishmania *(*Viannia*) *braziliensis *obtained from clinical samples of patients before and after treatment (typical lesions and scars, respectively) of the ACL.** Human lesions (black bars) and scars (gray bars).

**Figure 2:**
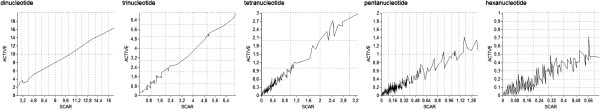
**Dinucleotide to hexanucleotide: distribution in *Leishmania *(*Viannia*) *braziliensis *minicircles obtained from clinical samples of patients before and after treatment (typical lesions and scars, respectively) of the ACL.**

The most frequent dinucleotides were AA, TT, AT and TA, ranging from 10 to 18% in frequency. Figure 1 shows that dinucleotide frequencies are slightly higher for sequences obtained from scars. A Wilcoxon signed rank test points to no significant difference for dinucleotide frequencies from active lesions and scar sequences, but for words above 3mer this value drops significantly (Table 3). Thus, at the dinucleotide level we cannot detect differences in minicircle sequence composition due to chemical pressure induced by the drug treatment. As expected from minicircle heterogeneity, for words of 3-, 4-, 5-, and 6mer the variation in frequency starts to be significant between an active lesion and a scar. The comparative analysis of minicircles poses the question of how their widely known sequence heterogeneity should be evidenced when the whole sequences are fragmented into their basic words. These words apparently have specific information, which in the case of trypanosomatids might be viewed as a sort of minicircle signature. By adding the composition of trinucleotides up to hexanucleotides it is possible to show the appearance of compositional heterogeneity with direct comparison of frequencies in both set of sequences, lesions and scars. Figure 2 summarizes this comparison at nucleotide length at the range 2- to 6mer in clinical samples for both sets (human lesions and scars). The graphics show the frequency variation as compared word by word after sorting the frequencies in decreasing order (sorting was fixed for the active set of sequences). This direct comparison is grounded in the fact that if no bias exists between the two sets, all the frequencies would display a straight line. Of course, as minicircle sequences are both multi-copies and extensively variable, we do observe some level of heterogeneity regardless of whether the sequence is partial or full length. This analysis shows the bias increasing along with the word length. For example, above the tetranucleotide level specific words start to appear at higher frequencies in one of the two sets. Zero frequency words are not counted for this graphical display, but it might be possible to show the nucleotide bias by counting words that are exclusively absent in both sets. The frequency graphics also show that words longer than 4mer are more sensitive to small variations in minicircle composition. This is expected from such an analysis because as we progress towards longer words, a single mutation in only one set can create a new word. Thus, larger fluctuations in frequency are observed in the lower part of the graphics, as can be observed for tetra- and pentanucleotides. On the other hand, increasing nucleotide word length also uncovers the zero frequency words, which lowers the interval of frequency variation. That is the case for 6mer (Figure 2). These observations imply that in the range 4- to 6mer, most of the nucleotide heterogeneity is represented by words of very low frequency. Overall, the graphics point that nucleotide word frequency from scar sequences is slightly lower than in active lesions.

**Table 3:** Wilcoxon signed rank test on frequency difference between active and scar lesion sequences at level of di, tri and tetranucleotide

**Probability**	**Same median**
Dinucleotide	1
Trinucleotide	0.65
Tetranucleotide	0.025

### Nucleotide words in the range 7–10mer: a cloud view

**Figure 3:**
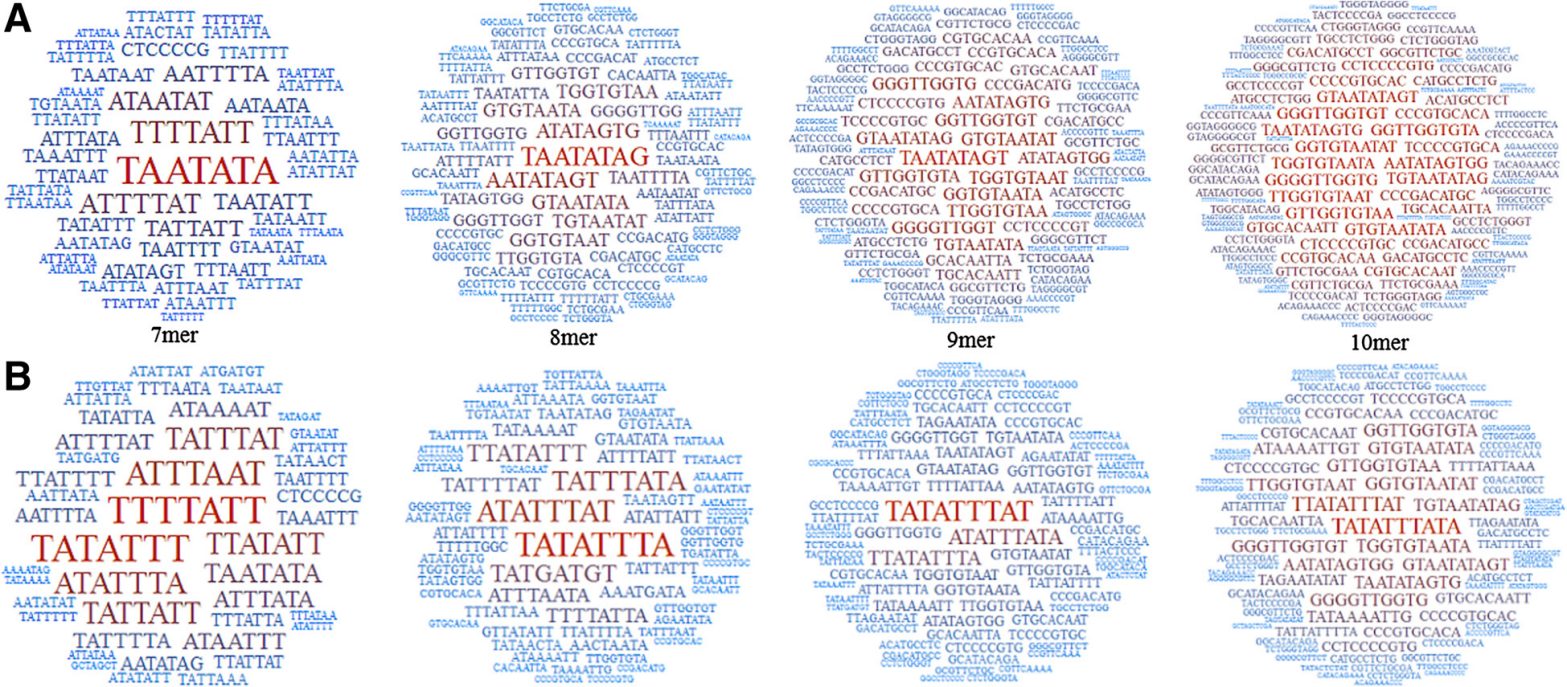
**Word cloud of nucleotide words found in minicircles from *Leishmania *(*Viannia*) *braziliensis *obtained from clinical samples of patients before and after treatment (typical lesions and scars, respectively) of the ACL.** Graphics represent the most frequent words composed of seven, eight, nine and ten nucleotides. **A)** active lesions; **B)** scars.

We analyzed the nucleotide words at the range 7- to 10mer in a population composed of kDNA minicircle sequences from *Leishmania* (*V*.) spp. The most frequent nucleotide words are graphically displayed using word cloud software, which allows a prompt visual grasping of the outstanding words for each set of sequences (Figure 3). The choice of this display method is based on the assumption that the partial set of sequences may represent the whole set of minicircles from this population (which we actually do not know). Thus, we expect that the most frequent nucleotide words might be a nucleotide sequence signature for all minicircles in each group. It is worth noting the relative dispersion of word frequency in both groups (Figure 3). The most frequent words from scar sequences are concentrated in a few words, in contrast to active sequences, which exhibit a higher number of words. The clouds show that the outstanding words (highest frequency) differ between active lesion and scar sequences. For example, the outstanding words for all lengths of the scar sequences are based on the core motif ATTT. In contrast, active sequences display a core motif (AATA) for 7- and 8mer and other motifs for words of 9- and 10mer. Though the most frequent words from both sets are composed mainly of A and T, in the composition of active sequences for 9- and 10mer words there is an increase of GT motifs. This is probably influenced by the presence of the universal minicircle sequence, GGGGTTGGTGTA, but we cannot discard the selection bias introduced by the PCR and subsequent molecular cloning. Regardless of the sequence being extracted, either from an active lesion or scar, the base T is the predominant one for the most frequent words in *Leishmania* (*V*.) spp. minicircles.

## Discussion

The minicircle sequences analyzed here were PCR-amplified as being of subgenus *Viannia* species directly from clinical samples (typical lesions and scars) of patients clinically cured of ACL in regions of endemism in Pernambuco state, Brazil [1]. It has been shown that *L*. *braziliensis* is the prevalent species to cause ACL in this region [1,32]. The specific PCR diagnostic carried out on these samples points to *Leishmania* (*V*.) spp. as the implicated species for the infection. *Leishmania* (*V*.) spp. DNA can be detected in scars [3-6] suggesting that persistence of parasites is the rule, rather than the exception, in leishmaniasis. As recent studies suggest that clinical cure of ACL is rarely associated with sterile cure [6], it is important to mention that parasite numbers present in scars is much lower than those in recent human lesions. Notwithstanding, the detection of *Leishmania* is high for this lesion [5,6]. We demonstrated here that sequences from either active lesions or scars do not show particular deviation from what has long been known to be the standard compositional bias for New World *Leishmania* minicircles. The extensive sequence of minicircles from both set offered new approaches to inspect peculiarities of sequence heterogeneity as well as the minicircle length variation from the same sample. This implies that the compositional repertoire of the *Leishmania* minicircles from clinical samples is dynamically variable and points to an unpredictable number of classes in each cell. Analysis of minicircles obtained from strains representing a unique trypanosomatid species showed that extensive polymorphisms are not uncommon [33-35]. Also, the diversity of *Leishmania* (*V*.) spp. populations [16,36] with their plethora of hosts, is contributing continuously to new sources of pressure on the parasites such as different immunological defenses, hostile environment and physico-chemical changes during the life cycle. Thus, a suitable approach to get information from variable sequences is to build a nucleotide word dictionary, which might be used as a marker for *Leishmania* spp. samples from endemic areas. To start with the compositional analysis we got the distribution of dinucleotides from both clinical samples. Though slight variation occurs between the two sets, it is not possible to attribute this small effect to an action of the therapeutic drug on minicircle composition. The high frequency of dinucleotides formed by adenines and thymines stems from the biased composition that has been observed in minicircles from New World *Leishmania*[16]. One of the questions that motivated this work is the shape of dinucleotide distribution upon physical or chemical pressure. *Leishmania* parasites have been naturally selected to survive in hostile environments, and considering that minicircle molecules are functionally redundant in the kDNA network [37] we could expect a relevant shift in their composition with the presence of a new pressure element-the drug used in leishmaniasis therapy. The data analysis performed in this work does not corroborate this assumption, and despite the mechanisms promoting the heterogeneity of minicircles, other factors may contribute to this relative compositional stability given the therapeutic chemical pressure. The profile in the composition of tri- up to hexanucleotides clearly shows an increase of variation of bases when increasing the number of nucleotide words, within the limits of the cutoff point. These findings may be related to the heterogeneity of kDNA, most likely to the different classes of minicircles observed in trypanosomatids in general.

## Conclusions

In summary, the analysis we presented here is a good approach to the development of a dictionary of nucleotide words based on minicircle sequences, and they might be useful for comparison of segments of *Leishmania* mitochondrial genome directly from human biological samples without the need for cultivation.

## Competing interests

The authors declare that they have no competing interests.

## Authors’ contributions

EHGR designed the experiments, performed the sequencing procedures, analyzed data, wrote the paper. FCSS extracted DNA, performed the PCR amplifications and cloning. RPW analyzed data. MEFB collected field samples, performed cultivation of *Leishmania* reference strain. OF conceived the study and designed the experiments. FGCA* conceived the study and designed the experiments. AB performed sequence analysis and statistics, analyzed data, wrote the paper. All authors read and approved the final version of the manuscript.

## References

[B1] Rodrigues EHG Brito MEF Mendonça MG Werkhäuser RP Coutinho EM Wayner VS Albuquerque MFM Jardim ML Abath FGC Evaluation of PCR for diagnosis of American cutaneous leishmaniasis in an area of endemicity in northeastern Brazil J Clin Microbiol 2002 40 3572 3576 10.1128/JCM.40.10.3572-3576.2002 12354848 PMC130853

[B2] Saravia NG Weigle K Segura I Giannini SH Pacheco R Labrada LA Gonçalves A Recurrent lesions in human *Leishmania braziliensis* infection-reactivation or reinfection? Lancet 1990 336 398 402 10.1016/0140-6736(90)91945-7 1974943

[B3] Guevara P Rojas E Gonzalez N Scorza JV Anez N Valera M Ramírez JL Presence of *Leishmania braziliensis* in blood samples from cured patients or at different stages of immunotherapy Clin Diagn Lab Immunol 1994 1 385 389 8556473 10.1128/cdli.1.4.385-389.1994 PMC368272

[B4] Aebischer T Recurrent cutaneous leishmaniasis: a role for persistent parasites? Parasitol Today 1994 10 25 28 15275563 10.1016/0169-4758(94)90353-0

[B5] Schubach A Haddad F Oliveira-Neto MP Degrave W Pirmez C Grimaldi Júnior G Fernandes O Detection of *Leishmania* DNA by polymerase chain reaction in scars of treated human patients J Infect Dis 1998 178 911 914 10.1086/515355 9728572

[B6] Mendonça MG Brito MEF Rodrigues EHG Bandeira V Jardim ML Abath FGC Persistence of *Leishmania* parasites in scars after clinical cure of American cutaneous leishmaniasis: is there a sterile cure? J Infect Dis 2004 189 1018 1023 10.1086/382135 14999605

[B7] Aebischer T Moody SF Handman E Persistence of virulent *Leishmania major* in murine cutaneous leishmaniasis: a possible hazard for the host Infect Immun 1993 61 220 226 8093358 10.1128/iai.61.1.220-226.1993 PMC302708

[B8] Rossel RA Duran RJ Rossel O Rodriguez AM Is leishmaniasis ever cured? Trans R Soc Trop Med Hyg 1992 86 251 253 10.1016/0035-9203(92)90297-P 1412645

[B9] Bogdan C Gessner A Solbach W Rollinghoff M Invasion, control and persistence of *Leishmania* parasites Curr Opin Immunol 1996 8 517 525 10.1016/S0952-7915(96)80040-9 8794010

[B10] Junqueira AC Degrave W Brandão A Minicircle organization and diversity in *Trypanosoma cruzi* populations Trends Parasitol 2005 21 270 272 10.1016/j.pt.2005.04.001 15922247

[B11] Osorio Y Gonzalez SJ Gama VL Travi BL Reinfection in American cutaneous leishmaniasis: evaluation of clinical outcomes in the hamster model Mem Inst Oswaldo Cruz 1998 93 353 356 9698870 10.1590/s0074-02761998000300015

[B12] Belkaid Y Hoffmann KF Mendez S Kamhawi S Udey MC Wynn TA Sacks DL The role of interleukin (IL)-10 in the persistence of *Leishmania major* in the skin after healing and the therapeutic potential of anti-IL-10 receptor antibody for sterile cure J Exp Med 2001 194 1497 1506 10.1084/jem.194.10.1497 11714756 PMC2193677

[B13] Bogdan C Rollinghoff M The immune response to *Leishmania*: mechanisms of parasite control and evasion Int J Parasitol 1998 28 121 134 10.1016/S0020-7519(97)00169-0 9504340

[B14] Karlin S Mrázek J Compositional differences within and between eukaryotic genomes Proc Natl Acad Sci USA 1997 94 10227 10232 10.1073/pnas.94.19.10227 9294192 PMC23344

[B15] Brewster S Barker DC Analysis of minicircle classes in *Leishmania* (*Viannia*) species Trans R Soc Trop Med Hyg 2002 96 55 63 10.1016/s0035-9203(02)90052-0 12055852

[B16] Chen KK Donelson JE Sequences of two kinetoplast DNA minicircles of *Trypanosoma brucei* Proc Natl Acad Sci USA 1980 77 2445 2449 10.1073/pnas.77.5.2445 6930643 PMC349416

[B17] Thiemann OH Maslov DA Simpson L Disruption of RNA editing in *Leishmania tarentolae* by the loss of minicircle-encoded guide RNA genes EMBO J 1994 13 5689 5700 7988566 10.1002/j.1460-2075.1994.tb06907.x PMC395534

[B18] Sturm NR Simpson L Kinetoplast DNA minicircles encode guide RNAs for editing of cytochrome oxidase subunit III mRNA Cell 1990 61 879 884 10.1016/0092-8674(90)90198-N 1693097

[B19] Simpson L The genomic organization of guide RNA genes in kinetoplastid protozoa: several conundrums and their solutions Mol Biochem Parasitol 1997 86 133 141 10.1016/S0166-6851(97)00037-6 9200120

[B20] Shu HH Stuart K Mitochondrial transcripts are processed but are not edited normally in *Trypanosoma equiperdum* (ATCC 30019) which has kDNA sequence deletion and duplication Nucleic Acids Res 1994 22 1696 1700 10.1093/nar/22.9.1696 8202373 PMC308051

[B21] Steinert M Van Assel S Sequence heterogeneity in kinetoplast DNA: reassociation kinetics Plasmid 1980 3 7 17 10.1016/S0147-619X(80)90030-X 7335823

[B22] Morel C Chiari E Camargo EP Mattei DM Roamanha AJ Simpson L Strains and clones of *Trypanosoma cruzi* can be characterized by pattern of restriction endonuclease products of kinetoplast DNA minicircles Proc Natl Acad Sci USA 1980 77 6810 6814 10.1073/pnas.77.11.6810 6256762 PMC350379

[B23] Spithill TW Grumont RJ Identification of species, strains and clones of *Leishmania* by characterization of kinetoplast DNA minicircles Mol Biochem Parasitol 1984 12 217 236 10.1016/0166-6851(84)90137-3 6090898

[B24] Langford CK Ullman B Landfear SM *Leishmania*: codon utilization of nuclear genes Exp Parasitol 1992 74 360 361 10.1016/0014-4894(92)90161-3 1582488

[B25] De Bruijn MH Barker DC Diagnosis of New World leishmaniasis: specific detection of species of the *Leishmania braziliensis* complex by amplification of kinetoplast DNA Acta Trop 1992 52 45 58 10.1016/0001-706X(92)90006-J 1359760

[B26] Sambrook J Fritsch EF Maniatis T Molecular cloning a laboratory manual 1989 New York: Cold Spring Harbor Laboratory Press

[B27] Kumar S Tamura K Nei M MEGA3 Integrated software for molecular evolutionary genetics analysis and sequence alignment Brief Bioinform 2004 5 150 163 10.1093/bib/5.2.150 15260895

[B28] Hammer O Harper DA Ryan PD PAST Paleontological statistics software package for education and data analysis Palaeont Electr 2001 4 9

[B29] Ray DS Conserved sequence blocks in kinetoplast minicircles from diverse species of trypanosomes Mol Cell Biol 1989 9 1365 1367 2542768 10.1128/mcb.9.3.1365-1367.1989 PMC362734

[B30] Rodriguez N Rodriguez A Cardona M Barrios MA McCann SH Barker DC *Leishmania* (*Viannia*) *guyanensis*: a new minicircle class exclusive to this specie isolated from a DNA cosmid library useful for taxonomic purposes Exp Parasitol 2000 94 143 149 10.1006/expr.1999.4482 10831378

[B31] Siegel S Castellan N Estatística não-paramétrica para as ciências do comportamento 2006 Rio de Janeiro: Artmed Editora

[B32] Brito ME Andrade MS Dantas-Torres F Rodrigues EH Cavalcanti MP Almeida AM Brandão-Filho SP Cutaneous leishmaniasis in northeastern Brazil: a critical appraisal of studies conducted in State of Pernambuco Rev Soc Bras Med Trop 2012 88 425 429 22836662 10.1590/s0037-86822012005000006

[B33] Rogers WO Wirth DF Generation of sequence diversity in the kinetoplast DNA minicircles of *Leishmania mexicana amazonensis* Mol Biochem Parasitol 1988 30 1 8 10.1016/0166-6851(88)90126-0 3398889

[B34] Fernandes O Catanho MP Segura I Labrada LA Derré R Saravia N Degrave W Minicircle variable region probes for characterization of *Leishmania* (*Viannia*) species J Parasitol 1999 85 563 568 10.2307/3285798 10386456

[B35] Pita-Pereira D Lins R Oliveira MP Lima RB Pereira BA Moreira OC Brazil RP Britto C SYBR Green-based real-time PCR targeting kinetoplast DNA can be used to discriminate between the main etiologic agents of Brazilian cutaneous and visceral leishmaniases Parasit Vectors 2012 12 1 9 10.1186/1756-3305-5-15 PMC3274473 22240199

[B36] Cupolillo E Brahim LR Toaldo CB de Oliveira-Neto MP Brito ME Falqueto A de Farias Naiff M Grimaldi Júnior G Genetic polymorphism and molecular epidemiology of *Leishmania* (*Viannia*) *braziliensis* from different hosts and geographic areas in Brazil J Clin Microbiol 2003 41 3126 3132 10.1128/JCM.41.7.3126-3132.2003 12843052 PMC165365

[B37] Simpson L Thiemann OH Savill NJ Alfonzo JD Maslov DA Evolution of RNA editing in trypanosome mitochondria Proc Natl Acad Sci USA 2000 97 6986 6993 10.1073/pnas.97.13.6986 10860961 PMC34374

